# Low-rank graph optimization for multi-view dimensionality reduction

**DOI:** 10.1371/journal.pone.0225987

**Published:** 2019-12-18

**Authors:** Youcheng Qian, Xueyan Yin, Jun Kong, Jianzhong Wang, Wei Gao

**Affiliations:** 1 Key Laboratory for Applied Statistics of MOE, School of Mathematics and Statistics, Northeast Normal University, Changchun, Jilin, China; 2 School of Science, Jilin Institute of Chemical Technology, Jilin, China; 3 School of Computer Science and Technology, Dalian University of Technology, Dalian, Liaoning, China; 4 School of Information Science and Technology, Northeast Normal University, Changchun, Jilin, China; Pavol Jozef Safarik University in Kosice, SLOVAKIA

## Abstract

Graph-based dimensionality reduction methods have attracted substantial attention due to their successful applications in many tasks, including classification and clustering. However, most classical graph-based dimensionality reduction approaches are only applied to data from one view. Hence, combining information from different data views has attracted considerable attention in the literature. Although various multi-view graph-based dimensionality reduction algorithms have been proposed, the graph construction strategies utilized in them do not adequately take noise and different importance of multiple views into account, which may degrade their performance. In this paper, we propose a novel algorithm, namely, Low-Rank Graph Optimization for Multi-View Dimensionality Reduction (LRGO-MVDR), that overcomes these limitations. First, we construct a low-rank shared matrix and a sparse error matrix from the graph that corresponds to each view for capturing potential noise. Second, an adaptive nonnegative weight vector is learned to explore complementarity among views. Moreover, an effective optimization procedure based on the Alternating Direction Method of Multipliers scheme is utilized. Extensive experiments are carried out to evaluate the effectiveness of the proposed algorithm. The experimental results demonstrate that the proposed LRGO-MVDR algorithm outperforms related methods.

## Introduction

In real-world applications, data can be represented by heterogeneous features. For example, images are often described by various types of features such as color, texture and shape. Web pages can also be represented in different styles, in which the text content and hyperlink information are two types of features. In these cases, each type of feature can be regarded as a particular view of data. Data represented by multiple types of features is referred to as multi-view data. In multi-view data, each individual view has its specific meaning to describe a particular aspect of data that cannot reflected by other views. However, different views also hold potential connection. Therefore, multiple views can be used to provide a complementary description of the data [1, 2]. Although the multi-view features are beneficial for distinguishing the samples of various classes, the feature vector of each view usually lies in a high-dimensional feature space and the combination of multi-view features typically results in the “curse of dimensionality” [3–5]. Therefore, it is essential to utilize dimensionality reduction technologies to reduce the feature redundancy among views while preserving most useful low-dimensional features.

In the past few decades, numerous dimensionality reduction methods have been developed for various tasks [2, 6–10]. These methods can be roughly categorized into two groups: linear and nonlinear [11, 12]. Linear dimensionality reduction methods map high-dimensional data to a suitable low-dimensional subspace via a linear projection. The classical linear dimensionality reduction methods are Principal Component Analysis (PCA) [13] and Linear Discriminant Analysis (LDA) [14]. The traditional linear dimensionality reduction algorithms are easy to implement because they only need to find an appropriate linear projection. However, the nonlinear structure of data cannot be exploited by them. Various nonlinear manifold-learning-based dimensionality reduction algorithms have been proposed to address this limitation, such as Locally Linear Embedding (LLE) [15], Isometric Feature Mapping (ISOMAP) [16], Laplacian Eigenmaps (LE) [17], Hessian Eigenmaps (HE) [18] and Local Tangent Space Alignment (LTSA) [19]. All the aforementioned dimensionality reduction algorithms are graph-based since they can be unified into a graph embedding framework [11]. However, these algorithms mainly focus on the features from a single view rather than multiple views.

A naive approach for multi-view feature embedding is simply to concatenate the features from various views as an input vector and apply dimensionality reduction methods on it directly. However, the concatenation strategy has several disadvantages: Firstly, the dimension of the concatenated vector is frequently high, which may cause the “curse of dimensionality” and lead to a high computational cost. Moreover, since each view describes a specific kind of information about the data, simply concatenating features of different views into a vector may not be physically meaningful. Furthermore, some research has shown that the performance of feature concatenation is worse than single view [20]. Recently, many algorithms have been proposed for constructing graphs by integrating information from multiple views. In [21], Xia et al. developed a new spectral embedding algorithm that learns a low-dimensional and sufficiently smooth embedding over all views simultaneously. In [20], Kumar and Daum applied co-training for multi-view data such that the graph in one view can be influenced the graph in the other view. In [22], Shu et al. constructed graphs separately from each view of the data and integrated multiple graphs by exploring higher-order information. In [23], Bisson and Grimal used multiple instances of an existing co-similarity approach to represent all the information in multi-view datasets simultaneously. By this way, their approach can transform the global data matrix into a graph. In [24], Tzortzis and Likas learned a weighted graph by minimizing the intra-cluster variance in the space induced by combining the individual graph of each view. In [25], De Sa et al. constructed a multipartite graph that was based on the minimizing-disagreement criterion [26]. In [27], Li et al. approximated the similarity graphs by using bipartite graphs. In [28], Zong et al. constructed a unified graph by learning local geometrical information in the original data space from multiple views simultaneously. However, none of the aforementioned algorithms has an explicit mechanism for handling potential noise and outliers in the data, which may degrade their performance.

Recently, some research showed that low-rank approximation is an effective method to remedy the influence of noise and outliers on graph [29]. As a result, many low-rank based algorithms have been developed to enhance the robustness of graphs to noise and outliers. In [30], Ye et al. proposed a robust late fusion method that decomposes each original graph from an individual model into a common rank-2 graph matrix and sparse deviation errors. In [31], Pan et al. proposed a robust rank aggregation algorithm that recovers a latent rank list from the possibly incomplete and noisy input rank lists. Similarly, Xia et al. [32] proposed a robust multi-view spectral clustering (RMSC) method that seeks a shared transition probability graph matrix with a low-rank constraint and a sparse error matrix. In [33], Hong et al. constructed a hypergraph Laplacian matrix by integrating various low-rank affinity graphs. Although these approaches have achieved satisfactory performance in dealing with noise, they neglected the different importance of multiple views and use the same weight for all input graphs. In fact, different views often contribute unequally in practice. Therefore, one challenge is how to aggregate the strengths of various heterogeneous graphs by exploring the rich information among them, which certainly can lead to more accurate and robust performance than by treating each individual type of graph equally [34].

We propose a novel algorithm, Low-Rank Graph Optimization for Multi-View Dimensionality Reduction (LRGO-MVDR) to address the aforementioned shortcomings. Compared with the existing methods, the proposed algorithm possesses two advantages. First, unlike the methods which ignore the noise and outliers in the datasets, our approach possesses a mechanism for dealing with noise and outliers based on a low-rank constraint. Thus, the features that obtained in our algorithm are more stable and robust. Second, different from most of the approaches which treat all views equally, a nonnegative weight vector is introduced into our algorithm to combine graphs from various views and an adaptive strategy is provided for optimizing the elements in the vector. Furthermore, we propose an optimization procedure based on the Alternating Direction Method of Multipliers (ADMM) scheme [35] to solve our model. We employ a simulated dataset and six real-world datasets to verify the performance of the proposed algorithm on classification and clustering tasks. Experimental results indicate that the proposed LRGO-MVDR algorithm outperforms other related algorithms in terms of classification accuracy rate [36, 37], Clustering Accuracy and Normalized Mutual Information [37, 38].

The remaining of the paper is organized as follows: Section ‘Related Work’ briefly reviews three methods for multi-view graph construction. Section ‘Proposed Algorithm’ describes the details of our algorithm. Section ‘Optimizing Algorithm’ describes the optimization process. Section ‘Experiments’ presents the experimental results and comparisons. Finally, Section ‘Conclusion and Future Work’ presents the conclusions and future work of this paper.

## Related work

An integral part of our method is the construction of an accurate and robust shared graph matrix by combining multiple graphs. In this section, three methods for multi-view graph construction will be reviewed.

### Robust late fusion with rank minimization

In [30], Ye et al. proposed a rank-based fusion method that utilizes rank minimization and sparse error to fuse the predicted confidence scores of multiple models, each of which is obtained based on a specified view of data. Given *m* confidence score vectors obtained from *m* views, which are denoted as ***s***^(1)^,***s***^(2)^,…,***s***^(*m*)^, where $s^{\left(i\right)}=\left[s_{1}^{\left(i\right)},s_{2}^{\left(i\right)},\ldots ,s_{n}^{\left(i\right)}\right]$, in which *n* is the number of samples, the method first converts each confidence score vector into a pairwise relationship matrix *T*^(*i*)^. Specifically, $T_{jk}^{\left(i\right)}=\mathrm{sign}\left(s_{j}^{\left(i\right)}-s_{k}^{\left(i\right)}\right)$, i.e., $T_{jk}^{\left(i\right)}=1$ if $s_{j}^{\left(i\right)}>s_{k}^{\left(i\right)}$, $T_{jk}^{\left(i\right)}=-1$ if $s_{j}^{\left(i\right)}<s_{k}^{\left(i\right)}$, and $T_{jk}^{\left(i\right)}=0$ if $s_{j}^{\left(i\right)}=s_{k}^{\left(i\right)}$, where $s_{j}^{\left(i\right)}$ and $s_{k}^{\left(i\right)}$ denote the scores of the *j*-th and *k*-th samples, respectively. The matrix *T*^(*i*)^ can encode the pairwise comparative relation of scores of every two samples under the *i*-th view. Each pairwise relationship matrix is combined with a shared graph matrix and an independent sparse residue matrix. The constrained optimization problem is formulated as follows:

$$\begin{array}{l} \underset{\hat{T},E^{\left(i\right)}}{\min}\mathrm{rank}\left(\hat{T}\right)+\lambda \sum_{i=1}^{m}{\left\|E^{\left(i\right)}\right\|}_{0},\\ s.t.T^{\left(i\right)}=\hat{T}+E^{\left(i\right)},\hat{T}=-{\hat{T}}^{T},i=1,\ldots ,m, \end{array}$$ (1)

where $\mathrm{rank}\left(\hat{T}\right)$ is the rank of $\hat{T}$, the *l*_0_-norm ${\left\|E^{\left(i\right)}\right\|}_{0}$ represents the number of non-zero elements in *E*^(*i*)^, and *λ* is a non-negative tradeoff parameter. In fact, *l*_0_-norm is not actually a norm, and the term ‘norm’ is used in this study for convenience [39].

Since the problem in Eq (1) is NP-hard, it is difficult to be solved. Recently, [40] proved that the nuclear norm function is the convex envelope of the rank function on the matrix unit sphere, so the nuclear norm is the best convex approximation of the rank function. More recently, it has been shown in [41–43] that the solution of the minimize rank can be obtained by solving the minimize nuclear problem. We also learned that the nuclear norm heuristic could produce very low-rank solutions in practice [40, 44] and corresponding theoretical characterization in [43]. In addition, *l*_1_-norm has been observed to be a good convex approximation to *l*_0_-norm [45]. Therefore, we could get a tractable optimization problem by minimizing the following convex optimization problem in Eq (2) instead of minimizing Eq (1). It is not difficult to see the minimization problems coincide each other with high probability,

$$\begin{array}{l} \underset{\hat{T},E^{\left(i\right)}}{\min}{\left\|\hat{T}\right\|}_{*}+\lambda \sum_{i=1}^{m}{\left\|E^{\left(i\right)}\right\|}_{1},\\ s.t.T^{\left(i\right)}=\hat{T}+E^{\left(i\right)},\hat{T}=-{\hat{T}}^{T},i=1,\ldots ,m. \end{array}$$ (2)

where the nuclear norm ${\left\|\hat{T}\right\|}_{*}$ denotes the sum of the singular values of $\hat{T}$, the *l*_1_-norm ${\left\|E^{\left(i\right)}\right\|}_{1}=\sum_{j=1}^{n}\sum_{k=1}^{n}\left|E_{jk}^{\left(i\right)}\right|$, *T*^(*i*)^ is a pairwise comparative relationship matrix, *E*^(*i*)^ represents the error matrix that corresponds to the *i*-th view, and $\hat{T}$ defines a shared graph matrix. The skew-symmetric constraint $\hat{T}=-{\hat{T}}^{T}$ is employed to make the decomposed $\hat{T}$ remain a pairwise comparative matrix. By minimizing the constrained optimization problem in Eq (2), a consistent shared graph among views can be discovered while overcoming the noise issues.

### Rank aggregation via low-rank and structured-sparse decomposition

In [31], Pan et al. stated that the method in [30] requires the input confidence score vectors to be complete (with no missing values), which is rare in practice. To overcome this shortcoming, a Robust Rank Aggregation (RRA) algorithm, which can simultaneously deal with the possible noise and missing values in the individual confidence score vector, was proposed. Given *m* confidence score vectors ***s***^(1)^,***s***^(2)^,…,***s***^(*m*)^ obtained from *m* views and $s^{\left(i\right)}=\left[s_{1}^{\left(i\right)},s_{2}^{\left(i\right)},\ldots ,s_{n}^{\left(i\right)}\right]$ in which *n* is the number of samples, RRA first converts each confidence score into a comparison matrix, which is denoted as *T*^(*i*)^. Specifically, $T_{jk}^{\left(i\right)}=\mathrm{sign}\left(s_{j}^{\left(i\right)}-s_{k}^{\left(i\right)}\right)$ if $s_{j}^{\left(i\right)}$ and $s_{k}^{\left(i\right)}$ are observed, and $T_{jk}^{\left(i\right)}=\mathrm{unknown}$ if $s_{j}^{\left(i\right)}$ or $s_{k}^{\left(i\right)}$ is missing, where $s_{j}^{\left(i\right)}$ and $s_{k}^{\left(i\right)}$ denote the scores of the *j*-th and *k*-th samples in the *i*-th view. The constrained optimization problem of a relaxation of RRA is defined as:

$$\begin{array}{l} \underset{Z,E^{\left(i\right)}}{\min}{\left\|Z\right\|}_{*}+\lambda \sum_{i=1}^{m}{\left\|E^{\left(i\right)}\right\|}_{2,1},\\ s.t.W^{\left(i\right)}⊙T^{\left(i\right)}=W^{\left(i\right)}⊙\left(Z+E^{\left(i\right)}-{\left(E^{\left(i\right)}\right)}^{T}\right),i=1,2,\ldots ,m, \end{array}$$ (3)

where the *l*_2,1_-norm regularization term ${\left\|E^{\left(i\right)}\right\|}_{2,1}=\sum_{k=1}^{n}\sqrt{\sum_{j=1}^{n}{\left(E_{j,k}^{\left(i\right)}\right)}^{2}}$ encourages the column-sparsity in *E*^(*i*)^, *λ* is a non-negative tradeoff parameter, ⊙ denotes elementwise (Hadamard) multiplication, *T*^(*i*)^ is a comparison matrix for the *i*-th view, *W*^(*i*)^ is an indicator matrix that corresponds to the *i*-th view such that $W_{jk}^{\left(i\right)}=0$ if $T_{jk}^{\left(i\right)}$ is unknown or missing and $W_{jk}^{\left(i\right)}=1$ otherwise, *Z* is the target graph matrix that encodes the true order relations among items, and *E*^(*i*)^ is used to encode the noise for the *i*-th view. From the definitions of *W*^(*i*)^ in Eq (3), it can be found that the objective function of RRA will not affected by the unknown or missing values in *T*^(*i*)^ since their corresponding elements in *W*^(*i*)^ is zero. Thus, as analyzed in [31], the main advantage of RRA over the method in [30] is that it can handle missing values.

### Robust multi-view spectral clustering via low-rank and sparse decomposition

Xia et al. [32] applied low-rank and sparse decomposition in a Markov chain and proposed an algorithm named Robust Multi-view Spectral Clustering via Low-rank and Sparse Decomposition (RMSC) for clustering. RMSC initially constructs a graph on each view and subsequently extracts a shared graph matrix using rank minimization. The constrained optimization problem of a relaxation of RMSC can be written as:

$$\begin{array}{l} \underset{\hat{P},E^{\left(i\right)}}{\min}{\left\|\hat{P}\right\|}_{*}+\lambda \sum_{i=1}^{m}{\left\|E^{\left(i\right)}\right\|}_{1},\\ s.t.\;P^{\left(i\right)}=\hat{P}+E^{\left(i\right)},\;\hat{P}\geq 0,\;\hat{P}1=1,i=1,2,\ldots ,m, \end{array}$$ (4)

where **1** denotes a column vector with all elements as 1, the *l*_1_-norm regularization term encourages the sparsity in *E*^(*i*)^, *λ* is a non-negative tradeoff parameter, *P*^(*i*)^ is a graph for the *i*-th view, $\hat{P}$ is a low-rank shared graph matrix and *E*^(*i*)^ is the error matrix for the *i*-th view. The constraints $\hat{P}\geq 0,\hat{P}1=1$ are employed to make $\hat{P}$ has a desired probability property, i.e., the optimal ${\hat{P}}_{ij}$ can be considered as a probability that the *i*-th and *j*-th samples are connected as a neighboring nodes in the graph [46, 47].

## Proposed algorithm

The methods in [30–32] are all graph-based multi-view methods and can effectively deal with noise. However, all views are treated equally and the diversity among multi-view data is not explicitly considered in these methods [30–32]. Therefore, their performance may suffer from the graph of less informative views [34]. To overcome this limitation, Low-Rank Graph Optimization for Multi-View Dimensionality Reduction (LRGO-MVDR) is proposed in this section, which consists of the following two steps.

### Shared graph matrix construction

**Fig 1:**
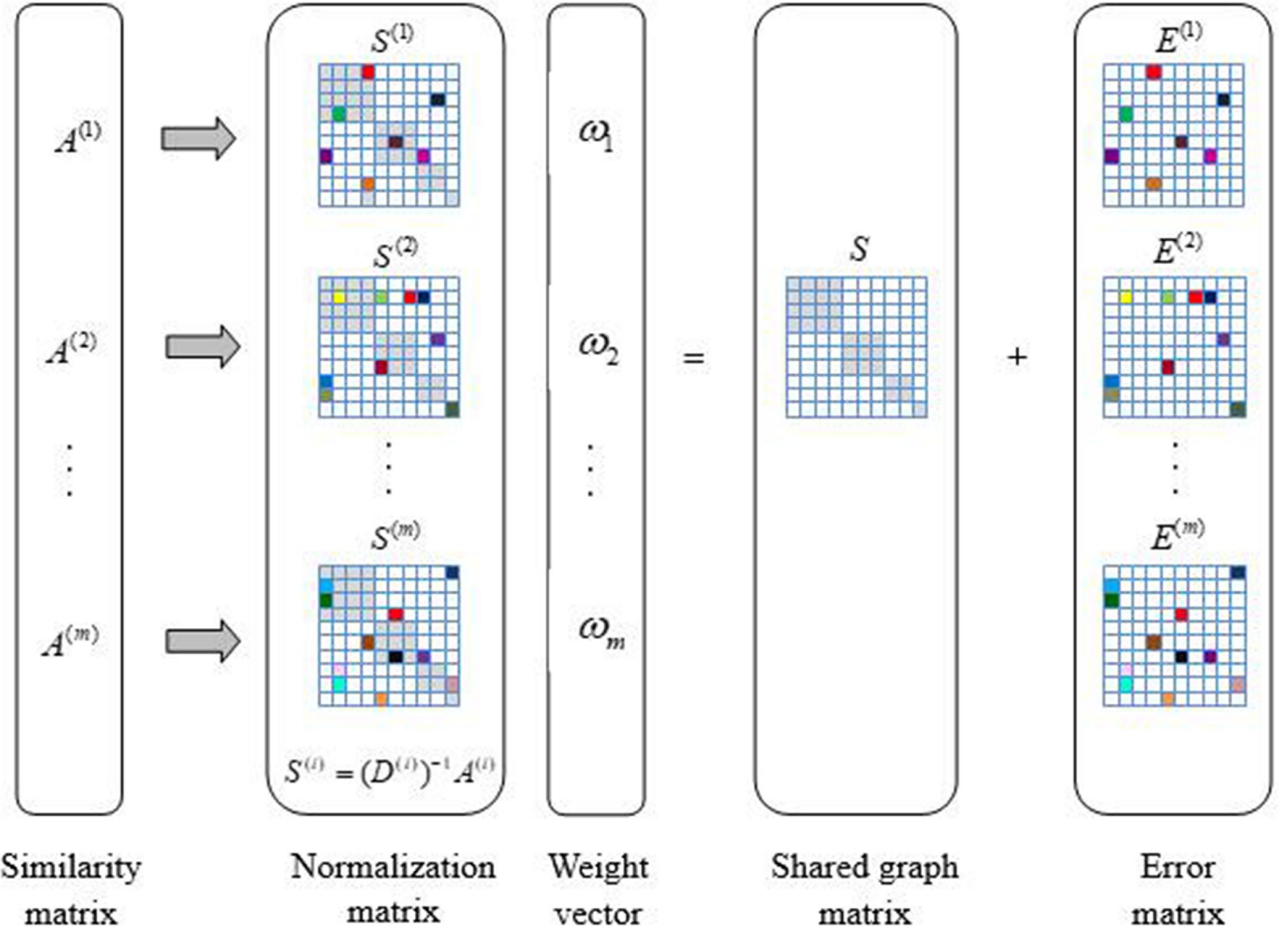
Overview of the shared graph matrix construction.

First, the graph of each view is constructed. Here, we also suppose *X* = {*X*^(1)^,*X*^(2)^,…,*X*^(*m*)^} is a multi-view dataset, where *m* is the number of views. $X^{\left(i\right)}=\left\{x_{1}^{\left(i\right)},\ldots ,x_{n}^{\left(i\right)}\right\}\in {\mathbb{R}}^{d_{i}\times n}$ is a set of *n* data points in the *i*-th view, which contains *c* classes. We define a similarity matrix $A^{\left(i\right)}\in {\mathbb{R}}^{n\times n}$ to represent the similarity between instances of the *i*-th view. Let *G*^(*i*)^ = (*V*^(*i*)^, *E*^(*i*)^, *A*^(*i*)^) be the weighted graph with vertex set *V*^(*i*)^, edge set *E*^(*i*)^, and similarity matrix *A*^(*i*)^. In general, the Gaussian kernel is used to define the similarity matrix:

$$A_{jk}^{\left(i\right)}=\exp\left(-\frac{{\left\|x_{j}^{\left(i\right)}-x_{k}^{\left(i\right)}\right\|}_{2}^{2}}{\sigma^{2}}\right),$$ (5)

where ${\left\|x_{j}^{\left(i\right)}-x_{k}^{\left(i\right)}\right\|}_{2}^{2}={\left(x_{j}^{\left(i\right)}-x_{k}^{\left(i\right)}\right)}^{T}\left(x_{j}^{\left(i\right)}-x_{k}^{\left(i\right)}\right)$ denotes the *l*_2_-norm and *σ*^2^ denotes average Euclidean distance over all pairs of data points. Let *S*^(*i*)^ be the normalization matrix of *G*^(*i*)^. We can define *S*^(*i*)^ as *S*^(*i*)^ = (*D*^(*i)*^)^−1^ *A*^(*i*)^, where *D*^(*i*)^ is a diagonal matrix with $D_{jj}^{\left(i\right)}=d_{j}^{\left(i\right)}=\sum_{k=1}^{n}A_{jk}^{\left(i\right)}$ and $d_{j}^{\left(i\right)}$ represents the degree of a vertex $v_{j}^{\left(i\right)}$ for the *i*-th view. Fig 1 illustrates the framework of our proposed method for the shared graph construction. There are two primary assumptions of LRGO-MVDR: (1) The features in each view are adequate for exploring most useful information. (2) The features in each view may be corrupted by noise. Based on these assumptions, each normalization matrix *S*^(*i*)^ when associated with an individual view can be naturally decomposed into two parts: a shared graph matrix *S* that reflects the underlying true structural information and an error matrix *E*^(*i*)^ that encodes the noise in each view.

$$\forall i,\;S^{\left(i\right)}=S+E^{\left(i\right)}.$$ (6)

### Problem formulation

To learn the final shared graph matrix *S* from each *S*^(*i*)^, we minimize the average disagreement between *S* and the cleaned matrices, i.e., *S*^(*i*)^−*E*^(*i*)^. Moreover, the normalization matrix from each view must be similar to the shared graph matrix. This requirement demands that the error matrices be small and sparse. It is formulated as follows:

$$\begin{array}{l} \underset{S,E^{\left(i\right)}}{\min}\sum_{i=1}^{m}{\left\|S-\left(S^{\left(i\right)}-E^{\left(i\right)}\right)\right\|}_{F}^{2}+\lambda {\left\|E^{\left(i\right)}\right\|}_{1},\\ s.t.\;S\geq 0,S1=1, \end{array}$$ (7)

where *λ* is a nonnegative tradeoff parameter. The *l*_1_-norm is used to ensure the sparsity of *E*^(*i*)^. The constraints *S*≥0,*S***1** = **1** enforce *S* to be nonnegative and the sum of each row to be 1.

**Fig 2:**
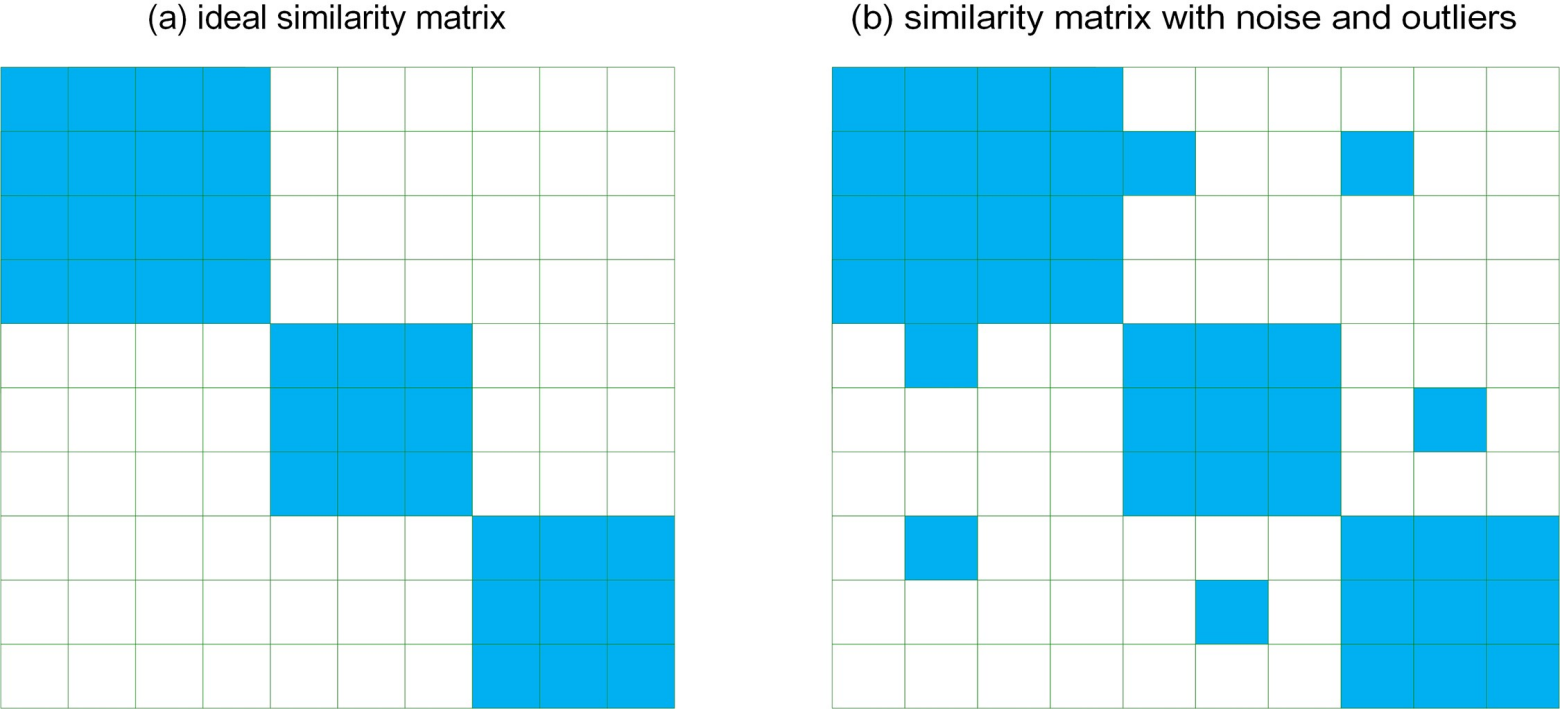
The examples of similarity matrices. The elements of zero values are illustrated with white and those of non-zero values are illustrated with blue.

Ideally, the similarity between two samples from the same class is high, whereas the similarity between the samples from different classes is low or even nearly zero. Therefore, as can be seen in Fig 2A, the similarity matrix in the ideal situation is diagonal block and its rank is low. However, the noise in the real-world data may affect the similarity of samples, e.g. make the similarity between samples from different classes be high, which will corrupt the diagonal block of similarity matrix and increase its rank (Fig 2B). Thus, the nuclear norm is introduced in our model to make the shared graph matrix *S* to be low rank, which would remedy the possible noise in the similarity matrices associated with different views and increase the robustness of our proposed approach. In summary, we can obtain the following formulation:

$$\begin{array}{l} \underset{S,E^{\left(i\right)}}{\min}\gamma {\left\|S\right\|}_{*}+\sum_{i=1}^{m}{\left\|S-\left(S^{\left(i\right)}-E^{\left(i\right)}\right)\right\|}_{F}^{2}+\lambda {\left\|E^{\left(i\right)}\right\|}_{1},\\ s.t.\;S\geq 0,S1=1, \end{array}$$ (8)

where ‖⋅‖_*_ denotes the nuclear norm of a matrix and *λ* is a nonnegative tradeoff parameter. Intuitively, no weight factor is explicitly defined in Eq (8) and all views are treated equally. Thus, the different importance of views is ignored. To further explore the complementarity and different importance of various views, a nonnegative weight vector denoted as $\omega ={\left[\omega_{1},\omega_{2},\ldots ,\omega_{m}\right]}^{T}\in {\mathbb{R}}^{m}$ is introduced in our approach, where *ω*_*i*_(*i* = 1,2,…*m*) is the weight for the *i*-th view. Thus, the Tikhonov regularization [48] term can be included and the final constrained optimization problem of our proposed LRGO-MVDR algorithm is:

$$\begin{array}{l} \underset{S,E^{\left(i\right)},\omega_{i}}{\min}\gamma {\left\|S\right\|}_{*}+\sum_{i=1}^{m}\omega_{i}\left({\left\|S-\left(S^{\left(i\right)}-E^{\left(i\right)}\right)\right\|}_{F}^{2}+\lambda {\left\|E^{\left(i\right)}\right\|}_{1}\right)+\beta {\left\|\omega \right\|}_{2}^{2},\\ s.t.\omega_{i}\geq 0,\sum \omega_{i}=1,S\geq 0,S1=1,i=1,\ldots ,m, \end{array}$$ (9)

where *β* is a nonnegative tradeoff parameter. The *l*_2_-norm regularization term $\|\omega {\|}_{2}^{2}$ is utilized to avoid overfitting of the weight vector *ω* to a single normalization matrix [49].

We can execute dimensionality reduction and obtain the low-dimensional representation by the shared graph matrix *S*, which will be discussed at Section ‘Dimensionality reduction with the shared graph’.

## Optimization algorithm

Since Eq (9) is not convex in all variables jointly, it is hardly to expect an algorithm to find its global optimal solution. However, due to Eq (9) is convex to in *S*, *E*^(*i*)^ and *ω*_*i*_ individually, the subproblem of solving each variable in this equation is tractable. We solve the problem via the Alternating Direction Method of Multipliers (ADMM) [35] scheme, which has demonstrated a satisfactory balance between efficiency and accuracy in many matrix learning problems [30–32].

By introducing an auxiliary variable *Q*, we convert Eq (9) into the following equivalent form:

$$\begin{array}{l} \underset{S,E^{\left(i\right)},\omega_{i},Q}{\min}\gamma {\left\|Q\right\|}_{*}+\sum_{i=1}^{m}\omega_{i}\left({\left\|S-\left(S^{\left(i\right)}-E^{\left(i\right)}\right)\right\|}_{F}^{2}+\lambda {\left\|E^{\left(i\right)}\right\|}_{1}\right)+\beta {\left\|\omega \right\|}_{2}^{2},\\ s.t.\;\omega_{i}\geq 0,\sum \omega_{i}=1,S\geq 0,S1=1,S=Q,i=1,\ldots ,m. \end{array}$$ (10)

The corresponding augmented Lagrangian function of Eq (10) is:

$$\begin{array}{l} L\left(S,Q,E^{\left(i\right)},\omega_{i}\right)=\gamma {\left\|Q\right\|}_{*}+\sum_{i=1}^{m}\omega_{i}\left({\left\|S-\left(S^{\left(i\right)}-E^{\left(i\right)}\right)\right\|}_{F}^{2}+\lambda {\left\|E^{\left(i\right)}\right\|}_{1}\right)+\beta {\left\|\omega \right\|}_{2}^{2}+\langle Y,S-Q\rangle +\frac{\mu }{2}{\left\|S-Q\right\|}_{F}^{2},\\ s.t.\;\omega_{i}\geq 0,\sum \omega_{i}=1,S\geq 0,S1=1,i=1,\ldots ,m, \end{array}$$ (11)

where 〈*P*,*S*−*Q*〉 = *tr*(*P*^*T*^(*S*−*Q*)) denotes the inner product of two matrices, *Y* represents the Lagrange multiplier, and *μ*>0 is an adaptive penalty parameter.

Next we will present the update rules for each of *S*, *Q*, *E*^(*i*)^ and *ω*_*i*_, which are obtained by minimizing *L* in Eq (11) with the other variables fixed.

**Optimize *Q* by fixing *S*, *E***^**(*i*)**^ **and *ω***_***i***_: Through fixing *S*, *E*^(*i*)^ and *ω*_*i*_, and removing the irrelevant terms, the optimization problem of *Q* can be obtained as:

$$\underset{Q}{\min}\gamma {\left\|Q\right\|}_{*}+\frac{\mu }{2}{\left\|S-Q+\frac{Y}{\mu }\right\|}_{F}^{2},$$ (12)

which can be solved via the Singular Value Threshold method [50]. More specifically, let *U*Σ*V*^*T*^ be the *SVD* form of (*S*+*Y*/*μ*), the solution of Eq (12) is as follows:

$$Q=US_{\gamma /\mu }\left(\Sigma \right)V^{T},$$ (13)

where *S*_*γ*/*μ*_(Σ) = max(Σ−*γ*/*μ*,0)+min(Σ+*γ*/*μ*,0) is the shrinkage operator [51].

**Optimize *E***^**(*i*)**^ **by fixing *Q*, *S* and *ω***_***i***_: Through fixing *Q*, *S* and *ω*_*i*_, and removing the irrelevant terms, the optimization of *E*^(*i*)^ becomes:

$$\underset{E^{\left(i\right)}}{\min}\lambda {\left\|E^{\left(i\right)}\right\|}_{1}+{\left\|S-\left(S^{\left(i\right)}-E^{\left(i\right)}\right)\right\|}_{F}^{2},$$ (14)

which has a closed-form solution of *E*^(*i*)^ = *S*_*λ*_(*S*^(*i*)^−*S*) according to [50] and *S*_*λ*_(*S*^(*i*)^−*S*) = max (*S*^(*i*)^−*S*−*λ*,0)+min(*S*^(*i*)^−*S*+*λ*,0) is the shrinkage operator [51].

**Optimize *S* by fixing *Q*, *E***^**(*i*)**^ **and *ω***_***i***_: When *Q*, *E*^(*i*)^ and *ω*_*i*_ are fixed, the optimization problem with respect to *S* becomes:

$$\begin{array}{l} S=\underset{S}{\arg\;\min}\sum_{i=1}^{m}\omega_{i}\left({\left\|S-\left(S^{\left(i\right)}-E^{\left(i\right)}\right)\right\|}_{F}^{2}\right)+\frac{\mu }{2}{\left\|S-Q+\frac{Y}{\mu }\right\|}_{F}^{2},\\ s.t.S\geq 0,S1=1. \end{array}$$ (15)

By simple algebraic formulation, we define:

$$C=\frac{1}{\sum_{i=1}^{m}\omega_{i}+\frac{\mu }{2}}\left(\sum_{i=1}^{m}\omega_{i}\left(S^{\left(i\right)}-E^{\left(i\right)}\right)+\frac{\mu }{2}Q-\frac{Y}{2}\right).$$ (16)

Then, Eq (15) is equivalent to

$$\begin{array}{l} S=\underset{S}{\arg\;\min}\left(\sum_{i=1}^{m}\omega_{i}+\frac{\mu }{2}\right){\left\|S-C\right\|}_{F}^{2}\\ \;=\underset{S_{1},\ldots ,S_{n}}{\arg\;\min}\left(\sum_{i=1}^{m}\omega_{i}+\frac{\mu }{2}\right)\sum_{i=1}^{n}{\left\|S_{i}-C_{i}\right\|}_{F}^{2},\\ s.t.\sum_{j=1}^{n}S_{ij}=1,S_{ij}\geq 0, \end{array}$$ (17)

where *S*_*i*_ and *C*_*i*_ denote the *i*-th rows of the matrices *S* and *C*, respectively. The problem in Eq (17) can be decomposed into *n* independent subproblems:

$$\underset{S_{i}}{\min}\left(\sum_{i=1}^{m}\omega_{i}+\frac{\mu }{2}\right){\left\|S_{i}-C_{i}\right\|}_{2}^{2},s.t.\sum_{j=1}^{n}S_{ij}=1,S_{ij}\geq 0.$$ (18)

Each subproblem is a projection of *C*_*i*_ onto the probability simplex, which can be efficiently solved via the projection algorithm in [52]. Here, we detail the algorithm in Algorithm 1.

Algorithm 1 The projection of a vector onto the probability simplex.

Input: A vector $C_{i}\in {\mathbb{R}}^{n}$

Sort *C*_*i*_ into *u*: *u*_1_≥*u*_2_≥⋯≥*u*_*n*_

Find $\hat{j}=\max\left\{j:1-\sum_{r=1}^{j}\left(u_{r}-u_{j}\right)\geq 0\right\}$

Let $\sigma =\left(\sum_{i=1}^{\hat{j}}u_{i}-1\right)/\hat{j}$

Output: *S*_*i*_, where *S*_*ij*_ = max(*C*_*ij*_−*σ*,0),*j* = 1,2,…,*n*

**Optimize *ω* by fixing *S*, *Q*, *E***^**(*i*)**^: When *S*, *Q* and *E*^(*i*)^ are fixed, we can rewrite Eq (11) with respect to *ω* as:

$$\begin{array}{l} \underset{\omega }{\min}\omega^{T}q+\beta {\left\|\omega \right\|}_{2}^{2},\\ s.t.\;\omega_{i}\geq 0,\sum \omega_{i}=1,i=1,\ldots ,m, \end{array}$$ (19)

where *q* = [*q*_1_,…,*q*_*m*_]^*T*^ and $q_{i}={\left\|S-\left(S^{\left(i\right)}-E^{\left(i\right)}\right)\right\|}_{F}^{2}+\lambda {\left\|E^{\left(i\right)}\right\|}_{1}$. In Eq (19), if *β* = 0, the trivial solution will be

$$\omega_{i}=\{\begin{array}{cc} 1, & \mathrm{if}\;q_{i}=\min_{{}_{k=1,2,\ldots ,m}}q_{k},\\ 0, & \mathrm{otherwise}. \end{array}$$ (20)

Since this situation only considers the information of a single view, the latent complementary information of multi-view datasets is ignored, which results in excessively sparse and unsatisfactory results. Contrarily, if *β*→∞, a uniform weight will be assigned to all multi-view datasets and the diversity of views will be neglected. Therefore, the best aggregated graph corresponds to *β* in the middle range value, i.e., a balance between averaged weighting and a single view.

The minimization in Eq (19) is a convex quadratic programming problem, which can be solved by the generic Lagrange multiplier and stochastic gradient decent methods [53]. However, for large-scale problems, these methods are usually time consuming and converge slowly. Thus, the Coordinate Descent Algorithm (CDA), which has very inexpensive iterations and can be easily parallelized, is employed in our algorithm to solve Eq (19) as suggested in [54–58]. The pseudocode of CDA is presented as Algorithm 2.

Algorithm 2 Coordinate Descent Algorithm (CDA)

1: Input: the tradeoff parameter *β*, the vector *q* = [*q*_1_,…,*q*_*m*_]

2: Output: the nonnegative weight vector *ω*

3: Initialize *ω*_*i*_ = 1/*m*,*i* = 1,2,…,*m*

4: for *i* = 1 to *m* do

5:   for *j* = 1 to *m*(*j*≠*i*) do

6:     repeat

7:       if 2*β*(*ω*_*i*_+*ω*_*j*_)+(*q*_*j*_−*q*_*i*_)≤0 then

8:         $\omega_{i}^{*}=0$, $\omega_{j}^{*}=\omega_{i}+\omega_{j}$

9:       else

10:         if 2*β*(*ω*_*i*_+*ω*_*j*_)+(*q*_*i*_−*q*_*j*_)≤0 then

11:             $\omega_{j}^{*}=0$, $\omega_{i}^{*}=\omega_{i}+\omega_{j}$

12:         else

13:             $\omega_{i}^{*}=\left(2\beta \left(\omega_{i}+\omega_{j}\right)+\left(q_{i}-q_{j}\right)\right)/\left(4\beta \right)$

14:             $\omega_{j}^{*}=\omega_{i}+\omega_{j}-\omega_{i}^{*}$

15:         end if

16:       end if

17:     until convergence

18:   end for

19: end for

### Complexity analysis for LRGO-MVDR

Our method LRGO-MVDR consists of four subproblems. The complexity of updating *Q* depends on the SVD of (*S*+*Y*/*μ*) and three matrix multiplications for *Q* = *US*_*γ*/*μ*_(Σ)*V*^*T*^, which is *O*(*n*^3^+*n*^3^) [50], where *n* is the sample number of data. For updating *E*^(*i*)^ in Eq (14) is *O*(*n*^2^), and for all *m* views, the complexity of *E* is *O*(*mn*^2^). For updating *S*, according to [52], we know that the complexity of *S* is *O*(*n*^2^log*n*). For updating *ω*, the main complexity is *O*(*tm*^2^) via Algorithm 2 [56]. Overall, the total complexity is *O*(*n*^3^+*n*^3^+*mn*^2^+*n*^2^log*n*+*tm*^2^) for each iteration. Under the condition *m*≪*n* and *t* is empirically set to 30, the total complexity is basically *O*(*Tn*^3^), where *T* is the number of iterations.

### Dimensionality reduction with the shared graph

Once we have obtained the shared graph matrix *S*, we can execute dimensionality reduction on it and obtain the low-dimensional representation. The constrained optimization problem is as follows:

$$\begin{array}{l} \;\frac{1}{2}\sum_{ij}{\left\|b_{i}-b_{j}\right\|}_{2}^{2}S_{ij}\\ =\frac{1}{2}\sum_{ij}\sum_{t=1}^{r}{\left(b_{it}-b_{jt}\right)}^{2}S_{ij}\\ =\sum_{t=1}^{r}B_{\cdot t}LB_{\cdot t}^{T}\\ =tr\left(BLB^{T}\right), \end{array}$$ (21)

where $B=\left[b_{1},b_{2},\ldots ,b_{n}\right]\in {\mathbb{R}}^{r\times n}$ is the low-dimensional representation of *X*^(*i*)^,*i* = 1,2,…,*m*, and *L* is the Laplacian matrix, which can be obtained as follows:

L=∏−S, (22)

where ∏ denotes a diagonal matrix of the shared graph matrix whose entries are the column or row sums of *S*. By introducing the constraint *B*Π*B*^*T*^ = *I*, the final constrained optimization problem is as follows:

$$\begin{array}{l} \underset{B}{\arg\;\min}\;tr\left(BLB^{T}\right),\\ s.t.\;B\Pi B^{T}=I. \end{array}$$ (23)

Eq (23) can be transformed into a generalized eigenvalue problem as:

$$LB^{T}=\alpha \prod B^{T}.$$ (24)

Suppose *α*_1_,*α*_2_,…,*α*_*r*_ are the *r* smallest eigenvalues of Eq (24) and *b*_1_,*b*_2_,…,*b*_*r*_ are the corresponding eigenvectors. The optimal low-dimensional features obtained by our algorithm is given by:

$$B={\left[b_{1},b_{2},\ldots b_{r}\right]}^{T}.$$ (25)

Finally, the overall optimization process of the LRGO-MVDR algorithm can be summarized in Algorithm 3. In Algorithm 3, the stop condition can be defined as convergence, i.e., ‖*S*−*Q*‖_∞_≤*ε*(*ε* = 10^-6^), or a prespecified maximum iteration number *T*_max_ (*T*_max_ = 300) is reached.

Algorithm 3 LRGO-MVDR algorithm

**Input:** the data *X*^(*i*)^(*i* = 1,2,…,*m*) and the parameters *γ*, *λ* and *β*

**Output:** the shared graph matrix *S*, noise matrices *E*^(*i*)^, weight vector *ω*, and the low-dimensional features *B*.

**Initialize**: *S* = 0, *Q* = 0, *Y* = 0, *μ* = 10^-3^, *ρ* = 1.1, max_*u*_ = 10^10^, *ω*_*i*_ = 1/*m*, *E*^(*i*)^ is a random matrix.

Compute similarity matrices *A*^(*i*)^ according to Eq (4) and compute the corresponding normalization matrices *S*^(*i*)^ via *S*^(*i*)^ = (*D*^(*i*)^)^−1^*A*^(*i*)^.

**Repeat**

1. Let $C\leftarrow \frac{1}{\sum_{i=1}^{m}\omega_{i}+\frac{\mu }{2}}\left(\sum_{i=1}^{m}\omega_{i}\left(S^{\left(i\right)}-E^{\left(i\right)}\right)+\frac{\mu }{2}Q-\frac{Y}{2}\right)$

2. for *j* = 1,2,…,*n*

3. Run Algorithm 1 using *C*_*j*_ as input to update *S*_*j*_, where *C*_*j*_/*S*_*j*_ is the *j*-th row of *C*/*S*

4. end for

5. Update *Q* via Eq (13)

6. for *i* = 1,2,…,*m*

7. Update *E*^(*i*)^ via Eq (14)

8. end for

7. Update *ω* according to Algorithm 2

8. Update *Y*←*Y*+*μ*(*S*−*Q*)

9. Update *μ*←min(*ρμ*,max_*μ*_)

**Until** stop condition is met

Obtain the low-dimensional features *B* via Eq (24).

### Convergence analysis

In this subsection, we analyze the convergence of the proposed LRGO-MVDR algorithm. According to the section of optimization, the optimization process of our algorithm can be divided into four subproblems, which are formulated in Eqs (12), (14), (15) and (19). According to [50], we know that there is a unique minimum in Eq (12), and the value of *E*^(*i*)^ decrease the function value of Eq (14). Following [52], we can solve for the minimum value of Eq (15). As for the subproblem in Eq (19), the objective function is obviously convex and then the minimization problem has unique solution. Thus, solving the four subproblems in Eqs (12), (14), (15) and (19) will decrease the value of our constrained optimization problem in each iteration [59]. In addition, since each term in Eq (9) is greater than zero, the constrained optimization problem of our LRGO-MVDR algorithm has a lower bound. Therefore, according to Monotonic Sequence Theorem, the proposed LRGO-MVDR algorithm will converge to a local minimum of Eq (9).

## Experiments

Since our LRGO-MVDR algorithm is a graph-based learning model, its performance is evaluated and compared with those of other related multi-view graph construction or optimization methods [20, 21, 23, 24, 32, 60, 61] on several benchmark datasets.

### Dataset description

**Fig 3:**
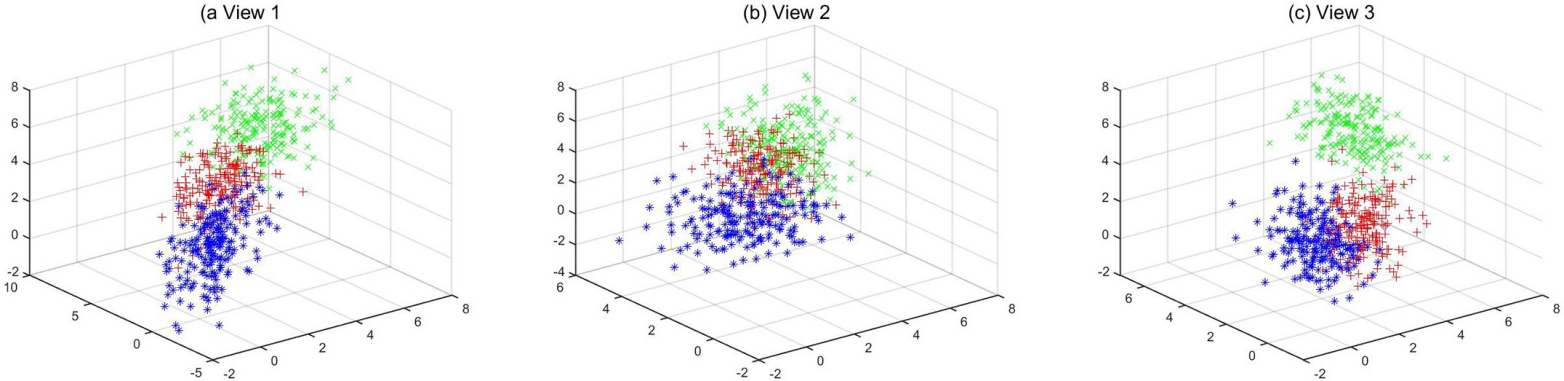
Simulated dataset example.

Simulated Dataset: Our simulated experiment is designed on a toy three-view data. In this dataset, 200 data points in each cluster of each view are sampled from a Gaussian distribution with three dimensions. The points in these clusters have diverse centers and covariances, which are overlapped and hence difficult to distinguish. Detailed information on this dataset is listed in Table 1, where *μ*_*ij*_ and ∑_*ij*_ represent mean and covariance of the *i*-th cluster in the *j*-th view, respectively. Fig 3 is the illustration of the simulated dataset.

**Table 1:** Detailed information on the simulated dataset.

		Cluster 1	Cluster 2	Cluster 3
View 1	Centers	*μ*_11_ = (1,1,1.2)	*μ*_21_ = (3,4,3)	*μ*_31_ = (5,5,5)
	Covariances	$\sum_{11}=\left(\begin{array}{ccc} 1 & 0.5 & 0.8\\ 0.5 & 1.5 & 0.6\\ 0.8 & 0.6 & 1.2 \end{array}\right)$	$\sum_{21}=\left(\begin{array}{ccc} 1.2 & 0.2 & 0.4\\ 0.2 & 0.9 & 0.1\\ 0.4 & 0.1 & 1.1 \end{array}\right)$	$\sum_{31}=\left(\begin{array}{ccc} 1 & ‐0.5 & 0.2\\ ‐0.5 & 1 & 0.3\\ 0.2 & 0.3 & 1 \end{array}\right)$
View 2	Centers	*μ*_12_ = (1,2,1.4)	*μ*_22_ = (3,3,3)	*μ*_32_ = (4,3,4)
	Covariances	$\sum_{12}=\left(\begin{array}{ccc} 1.2 & ‐0.2 & 0.3\\ ‐0.2 & 1.3 & ‐0.4\\ 0.3 & ‐0.4 & 1.5 \end{array}\right)$	$\sum_{22}=\left(\begin{array}{ccc} 0.6 & 0.1 & 0.2\\ 0.1 & 0.9 & 0.3\\ 0.2 & 0.3 & 1 \end{array}\right)$	$\sum_{32}=\left(\begin{array}{ccc} 0.8 & 0.1 & 0.2\\ 0.1 & 0.9 & 0.3\\ 0.2 & 0.3 & 1.5 \end{array}\right)$
View 3	Centers	*μ*_13_ = (1,1,2)	*μ*_23_ = (3,1,2)	*μ*_33_ = (5,4,5)
	Covariances	$\sum_{13}=\left(\begin{array}{ccc} 0.6 & 0.2 & 0.3\\ 0.2 & 1 & 0.4\\ 0.3 & 0.4 & 1 \end{array}\right)$	$\sum_{23}=\left(\begin{array}{ccc} 1 & 0.4 & 0.2\\ 0.4 & 0.7 & 0.3\\ 0.2 & 0.3 & 0.9 \end{array}\right)$	$\sum_{33}=\left(\begin{array}{ccc} 1 & 0.4 & ‐0.2\\ 0.4 & 1 & 0.3\\ ‐0.2 & 0.3 & 0.9 \end{array}\right)$

Caltech101 Dataset [62]: This is an object recognition dataset that contains 101 categories of images. We select 11 widely used classes, namely, dollar-bill, anchor, ant, cougar-body, elephant, flamingo, panda, platypus, seahorse, snoopy, and wildcat, and obtain 512 images. Three types of features are extracted to represent each image: 512-dimensional GIST features, 254-dimensional CENTRIST features and 36-dimensional LBP features.

Wiki Dataset [63]: This is a widely used dataset for cross-modal retrieval, which consists of 693 image-text pairs that are divided into 10 categories. In each pair, the image is encoded by 128-dimensional SIFT descriptors and the text consists of 10-dimensional topics that are derived from a Latent Dirichlet Allocation model.

Yale Dataset [64]: The Yale face dataset contains 165 grayscale images of 15 individuals. There are 11 images per subject and each has a different facial expression or configuration. We extract three types of features: 9-dimensional color moment, 50-dimensional LBP and 512-dimension GIST features.

Cornell Dataset [65]: This dataset is composed of web pages that were collected from the computer science department of Cornell University. There are 195 pages over 5 labels (student, project, course, staff, faculty). Two heterogeneous feature sets, namely, cites and content, are utilized here for experiments. Specifically, the pages are described by 1703 words in the content view, which is a sparse matrix containing 0 and 1 values indicating absence and presence of a word in a page; and the number of citation links between other pages in the cites view.

Wisconsin Dataset [65]: This dataset is composed of web pages that were collected from the computer science department of the University of Wisconsin. The archive contains 265 documents over 5 labels. Two views are considered for each webpage: content and cites. The detailed description is similar to the Cornell dataset.

WebKB Dataset [66]: This dataset contains a subset of the web pages that were collected from computer science departments of various universities in January 1997 by the World Wide Knowledge Base project of the CMU text learning group. The 1051 pages were manually classified into two categories. Each webpage can be described by content view and cites view. The detailed description is similar to the Cornell dataset.

Data preprocessing is used for all datasets. Specifically, each feature variable in the simulated data set and the six real-world datasets have been transformed to have sample mean 0 and variance 1.

### Compared algorithms

BSV [67]: This algorithm performs tasks independently on each view and chooses a view that achieves the best performance.

FeaConc [32]: This algorithm concatenates the features from each view and performs subsequent tasks directly on this concatenated feature representation.

KA [32]: This algorithm constructs a kernel matrix for the data from each view and sums these matrices over all views to obtain a single kernel matrix.

MVSim [23]: This algorithm simultaneously deals with all the information that is contained in multi-view datasets by using several instances of an existing co-similarity algorithm.

MVSpec [24]: This algorithm learns a weighted combination of the specified kernels in parallel.

MvSpecCE [61]: This algorithm extends clustering ensembles to multi-view clustering.

Cospectral [20]: This algorithm constructs a graph in one view by using the eigenvectors in another view and the tasks are subsequently performed based on the graph.

Corespectral [60]: This algorithm regularizes the eigenvectors of view-dependent graph Laplacians and a pairwise co-regularization scheme is used in our experiment.

MSE [21]: This algorithm combines features into a feature matrix and uses matrix decomposition methods to obtain a low-dimensional embedding matrix.

RMSC [32]: This algorithm recovers a shared graph matrix via low rank and sparse decomposition.

All experiments in this work are implemented in MATLAB R2018b and run on an Intel Core i7-8700K CPU at 3.70 GHz with 16 GB physical memory.

### Clustering

In this section, the performance of the proposed algorithm on clustering task is evaluated on four datasets: Simulated, Wisconsin, WebKB and Cornell. Information on these datasets is listed in Table 2.

**Table 2:** Detailed information on the datasets that are used for clustering.

Dataset	Size	Views	Clusters
Simulated	600	3	3
Wisconsin	265	2	5
WebKB	1051	2	2
Cornell	195	2	5

#### Experimental settings

We set the values of parameters in the comparison algorithms according to the experiments in their corresponding literature [20, 21, 23, 24, 32, 60, 61]. In our approach, we empirically tune the values of parameters *γ* and *λ* in the range {10^-4^,10^-3^,…,10^5^} and we tune *β* by searching the grid {0.01,0.02,0.03,0.04,0.05,0.06,0.07,0.08,0.09,0.1,0.2,0.3,0.4,0.5,0.6,0.7,0.8,0.9,1} in an alternating manner [54].

For the clustering task, we use two types of evaluation metrics to measure the performance: Clustering Accuracy (ACC) and Normalized Mutual Information (NMI) [37, 38]. The Clustering Accuracy is defined as follows:

$$\mathrm{ACC}=\frac{\sum_{i=1}^{n}\sigma \left(l_{i},map(c_{i})\right)}{n},$$ (26)

where *σ*(*x*,*y*) = 1 if *x* = *y* and *σ*(*x*,*y*) = 0 otherwise, *n* is the total number of samples, *l*_*i*_ is the true class label of the *i-*th sample, *c*_*i*_ is the clustering result label of the *i*-th sample, and *map*(⋅)is a best mapping function that uses the Kuhn-Munkres algorithm to map clustering labels to equivalent ground-truth labels [68]. NMI is defined as follows:

$$\mathrm{NMI}=\frac{I\left(L,C\right)}{\sqrt{H\left(L\right)H\left(C\right)}},$$ (27)

where *I*(*L*,*C*) denotes the mutual information between true class label *L* and obtained cluster label *C*, *H*(*L*) and *H*(*C*) are the entropies of *L* and *C*. According to Eqs (26) and (27), a larger value of ACC or NMI indicates a better clustering performance. Moreover, the *k*-means algorithm is adopted to perform clustering on the low-dimensional features obtained by different approaches. Because the performance of *k*-means is heavily dependent on the initialization, we repeat the experiment 10 times with random initialization. The average accuracies and the corresponding standard deviations are recorded in the following section.

#### Experimental results and analysis

**Fig 4:**
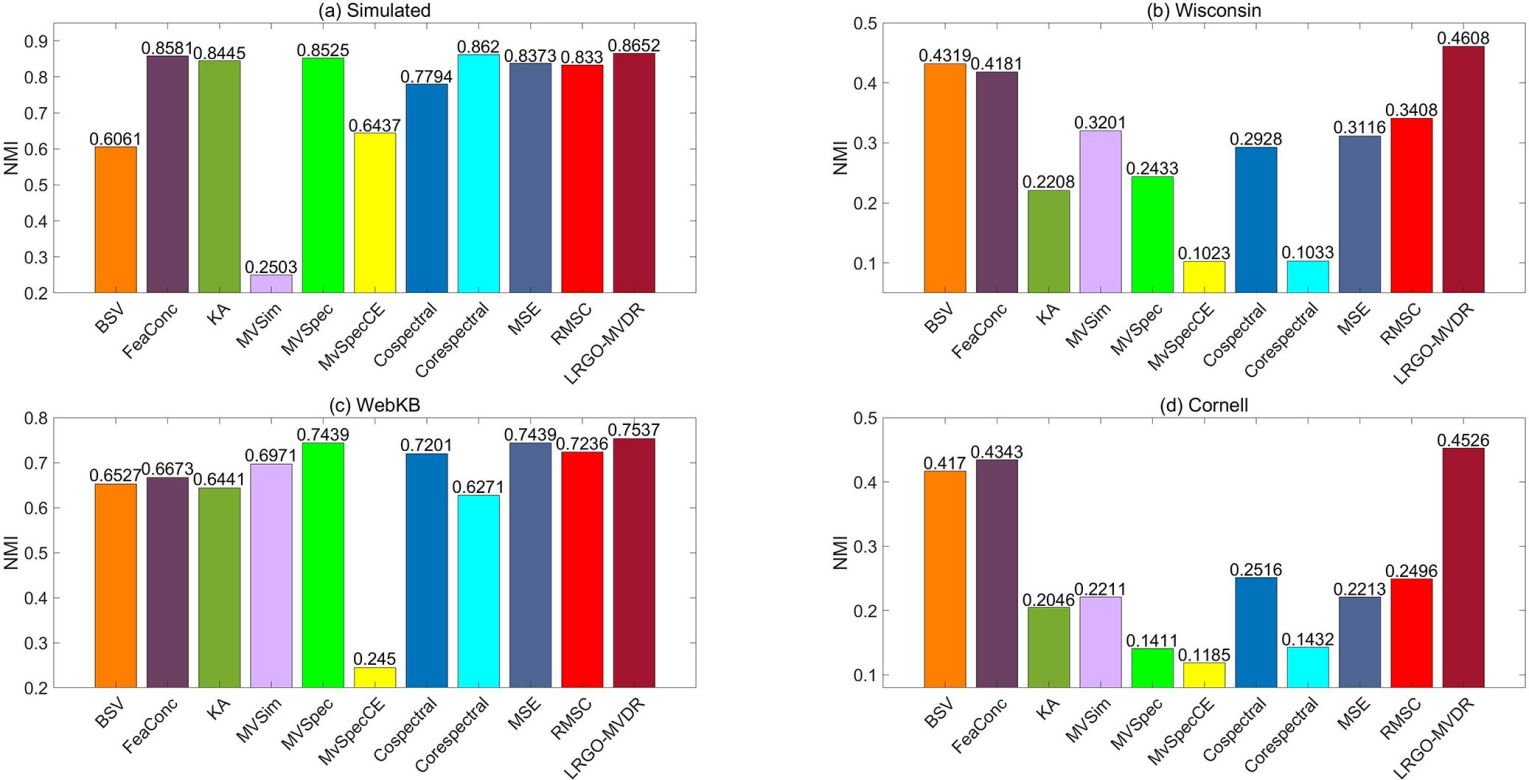
NMI results of various algorithms.

The highest clustering accuracies obtained by various algorithms are listed in Table 3. According to this table, FeaConc outperforms BSV in most cases, which demonstrates that multi-view data can provide more complementary information. Although RMSC [32] has a mechanism for dealing with noise, our approach outperforms it. This is because RMSC treats all views equally, which is not appropriate since the differences among views are ignored. Moreover, different algorithms employ different ways to fuse graphs. Nevertheless, their results are inferior to those of our method. This is because our method adequately takes noise and different importance among multiple views into consideration. Last, the proposed LRGO-MVDR algorithm outperforms all the other algorithms; the best NMI results obtained by the algorithms are shown in Fig 4.

**Table 3:** Best clustering results (ACC±std) of various algorithms.

Methods	Simulated	Wisconsin	WebKB	Cornell
BSV	0.8295±0.0048 (2, 1.2883)	0.6298±0.0254 (4, 2.0602)	0.9519±0.0784 (2, 5.7666)	0.5205±0.0373 (4, 2.8671)
FeaConc	0.9583±0.0000 (2, 0.7068)	0.6049±0.0428 (4, 1.3095)	0.9429±0.0030 (2, 3.3374)	0.5477±0.0349 (4, 1.7544)
KA	0.9540±0.0008 (2, 0.7436)	0.4668±0.0362 (10, 1.1770)	0.9353±0.0000 (2, 3.3640)	0.4364±0.0045 (4,1.7907)
MVSim	0.6510±0.0052 (2, 1.6887)	0.5868±0.0621 (2, 3.7267)	0.9479±0.0064 (7, 15.6147)	0.4626±0.0153 (2, 4.6397)
MVSpec	0.9560±0.0016 (2, 1.1547)	0.4438±0.0222 (4, 2.2537)	0.9667±0.0000 (3, 22.8464)	0.4113±0.0164 (3, 3.1782)
MvSpecCE	0.8700±0.0000 (2, 60.3064)	0.4053±0.0184 (6, 201.025)	0.7275±0.0512 (6, 5118.3)	0.4267±0.0093 (2, 439.42)
Cospectral	0.9283±0.0000 (2, 6.1690)	0.5457±0.0164 (4, 14.3779)	0.9600±0.0000 (3, 79.8809)	0.4374±0.0123 (16, 21.1995)
Corespectral	0.9600±0.0000 (2, 2.5740)	0.4706±0.0199 (6, 7.7287)	0.9365±0.0005 (3, 41.8010)	0.4318±0.0203 (4, 10.3666)
MSE	0.9517±0.0000 (2, 1.3142)	0.5189±0.0368 (6, 1.7598)	0.9667±0.0000 (3, 19.0447)	0.4621±0.0294 (4, 2.4776)
RMSC	0.9497±0.0004 (2, 7.3515)	0.5381±0.0211 (6, 2.0915)	0.9667±0.0000 (3, 26.0721)	0.4718±0.0331 (15, 2.2652)
LRGO-MVDR	**0.9613±0.0007 (2, 3.9956)**	**0.6415±0.0205 (6, 1.8747)**	**0.9686±0.0000 (3, 18.2413)**	**0.6026±0.0081 (4, 2.3279)**

The numbers in parentheses are the feature dimension corresponds to the best result and the training time of each algorithm, respectively.

**Fig 5:**
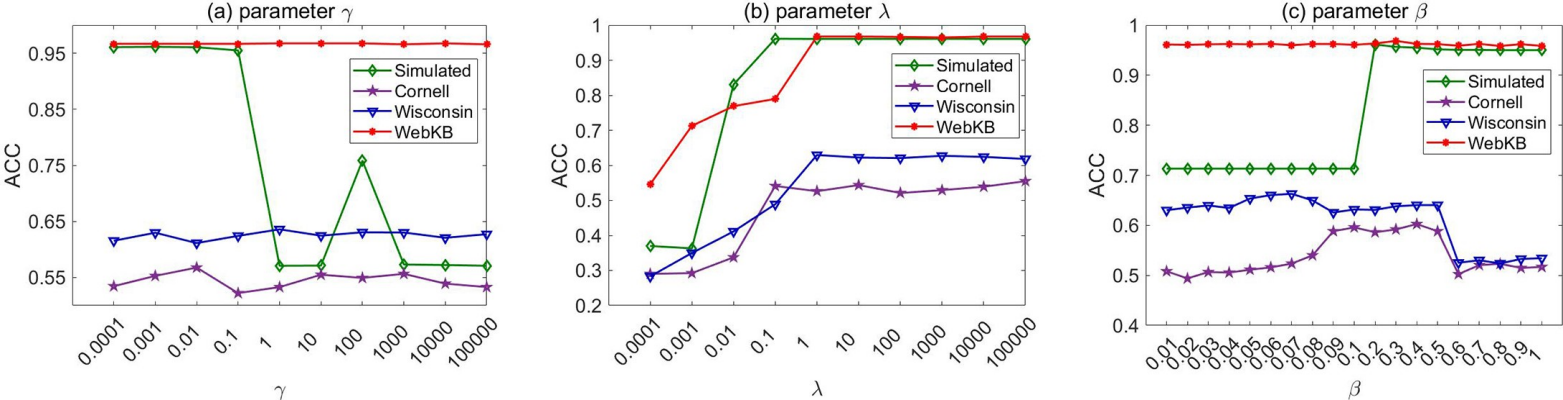
Performance of LRGO-MVDR under various parameter values for clustering tasks on four datasets.

Next, the performance of our LRGO-MVDR algorithm under various parameter values (*γ*, *λ* and *β*) is evaluated. According to the experimental results in Fig 5A, the performance of our method is stable across a wide range of *γ* values (especially for the experiments on the Wisconsin, WebKB and Cornell datasets). According to the results in Fig 5B, when the value of *λ* is very small, the results that are obtained by LRGO-MVDR are relatively poor. With the increase of the value of *λ*, the performance of our algorithm improves significantly. This indicates that considering the noise in the data plays an important role in the proposed algorithm. Moreover, when *λ* exceeds a specified value, the influence of *λ* on our algorithm becomes small; hence, a larger *λ* is preferable for the proposed LRGO-MVDR algorithm. In Fig 5C, we find that *β* influences the performance of our algorithm. As we have analyzed, our algorithm performs better when *β* is set as a moderate value since a small *β* will make our LRGO-MVDR overlook the latent complementary information of multi-view data while a large *β* will lead the different importance of views to be ignored. In general, the best *β* value for our algorithm is highly data-dependent, but we can roughly observe that the satisfactory results are produced in the range of [0.05,0.5].

**Fig 6:**
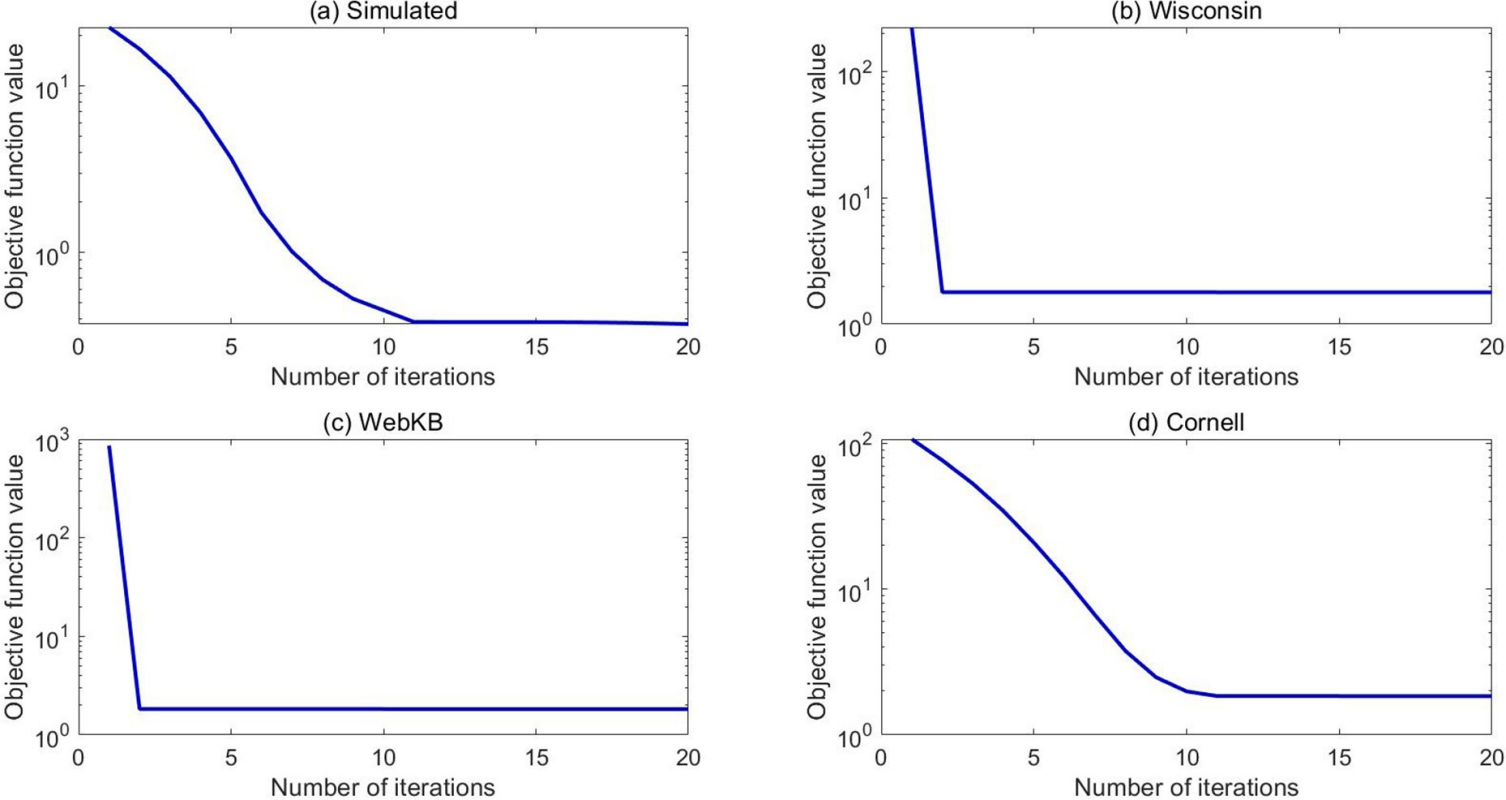
Convergence curves of LRGO-MVDR on four datasets for clustering.

Then, the convergence curves of our LRGO-MVDR algorithm on four datasets are shown in Fig 6. In this figure, the x-axis and logarithm y-axis represent the iteration number and the value of Eq (9), respectively. Fig 6 demonstrates that the value of Eq (9) monotonically decreases at each iteration. Here, we should point out that although the curves in Fig 6B and Fig 6C seem to be flat after only a few (i.e., 1–2) iterations, it actually keeps decreasing. Since the value changes of Eq (9) in these two cases are relatively small after 1–2 iterations, it is hard to observed from the figure.

Finally, in order to justify the choice of coordinate descent algorithm in our approach, we compare the performance of LRGO-MVDR with the same model which utilizes Lagrange multiplier and stochastic gradient descent methods for solving Eq (19). Here, we use LRGO-MVDR-C, LRGO-MVDR-L and LRGO-MVDR-S to denote the proposed LRGO-MVDR which adopts coordinate descent, Lagrange multiplier and stochastic gradient descent methods to optimize Eq (19), respectively. From the experimental results in Table 4, it can be found that LRGO-MVDR-C and LRGO-MVDR-L achieve very similar clustering performances. This may because that they converge to the nearly identical solutions during the optimization. However, due to the efficiency of coordinate descent algorithm, the training time of LRGO-MVDR-C is less than LRGO-MVDR-L. Since stochastic gradient descent needs to compute the gradient of Eq (19) and solve it along the negative of gradient, the convergence speed of LRGO-MVDR-S is slower than LRGO-MVDR-C and LRGO-MVDR-L. Moreover, we can also see that the clustering performance of LRGO-MVDR-S is inferior to other two approaches, which may due to it cannot find the optimum solution when the maximum number of iterations is reached.

**Table 4:** Best clustering results (ACC±std) of three methods for solving Eq (19).

Methods	Simulated	Wisconsin	WebKB	Cornell
LRGO-MVDR-L	0.96129239±0.0007 (2, 4.1361)	0.64145186±0.0205 (6, 2.4216)	0.96857136±0.0000 (3, 18.4467)	0.60262186±0.0081 (4, 2.8211)
LRGO-MVER-S	0.95758912±0.0008 (2, 12.1196)	0.63234186±0.0116 (6, 6.6156)	0.96574672±0.0000 (2, 39.8058)	0.59854761±0.0321 (4, 5.1145)
LRGO-MVDR-C	**0.96132768±0.0007 (2, 3.9956)**	**0.64152481±0.0205 (6, 1.8747)**	**0.96859271±0.0000 (3, 18.2413)**	**0.60264173±0.0081 (4, 2.3279)**

The numbers in parentheses are the feature dimension corresponds to the best result and the training time of each algorithm, respectively.

### Classification

In this section, we use four publicly available datasets, namely, Caltech101, Wiki, Yale and Cornell, to assess the performance of the proposed approach on classification tasks. Detailed information on the datasets is tabulated in Table 5.

**Table 5:** Detailed information on the datasets for classification.

Dataset	Size	Views	Classes	*l*	*t*
Caltech101	512	3	11	258	254
Wiki	693	2	10	349	344
Yale	165	3	15	90	75
Cornell	195	2	5	99	96

#### Experimental settings

For classification task, we repeated 10 times of hold-out validation for each dataset to evaluate the performance [69]. For each hold-out validation, each data set is randomly split into two sets of sizes *l* and *t*, the first is treated as the training data, and the other part is test data. The values of *l* and *t* for various datasets are listed in Table 5. Then we obtain an average accuracy rate of 10 repetitions as hold-out validated accuracy (HVA). In this task, the nearest-neighbor classifier is used to measure each method’s performance due to its simplicity. The HVA is defined as follows:

$$\mathrm{HVA}=\frac{1}{h}\sum_{i=1}^{h}\frac{N_{cor}^{\left(i\right)}}{N_{total}},$$ (28)

where *h* is the times of hold-out validation, $N_{cor}^{\left(i\right)}$ is the number of test samples that are correctly classified in *i*-th time hold-out validation by the nearest-neighbor classifier and *N*_*total*_ is the total number of test samples [36, 37]. We report the highest HVA for all of parameter sets of (*γ*,*λ*,*β*).

#### Experimental results and analysis

**Fig 7:**
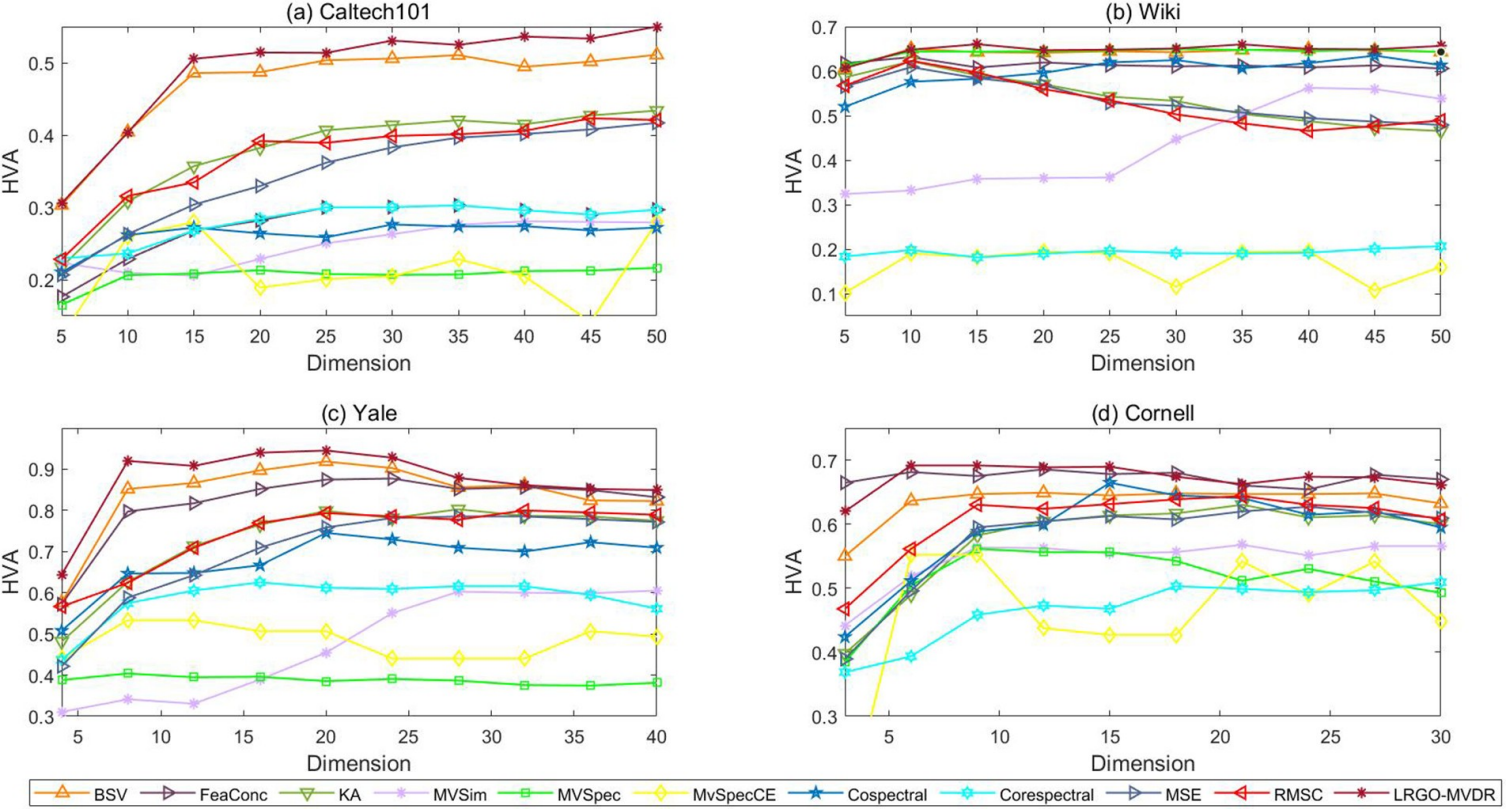
Classification performance of various algorithms.

Fig 7 shows the classification accuracy comparison among the algorithms. In this figure, the x-axis and y-axis represent the low-dimensional representation dimensions and HVA, respectively. From Fig 7, we observe the following points: First, the performance of our algorithm is basically consistent with those of BSV which reports the best results that are achieved using a single view. However, choosing the view that yields the optimal recognition performance in practical applications is time consuming and computationally costly. Thus, adaptively combining graphs that are obtained by multiple views is important. Second, our method becomes more stable than other algorithms with dimension varies. Specifically, the HVA of our algorithm improves steadily with the increase of dimension, and fluctuates less than some other algorithms. Hence, the proposed method is more robust. Third, our proposed LRGO-MVDR algorithm outperforms other algorithms that treat all graphs equally, such as FeaConc, KA, MVSim, Cospectral, Corespectral and RMSC, which demonstrates the effectiveness of introducing the weighted vector for the various graphs in our algorithm. Furthermore, the HVA obtained by different algorithms in Table 6 can also demonstrate the advantage of our algorithm. Last, the proposed LRGO-MVDR algorithm outperforms all the other algorithms. This is attributed to LRGO-MVDR explicitly taking noise and differences in importance among the data of multiple views into consideration.

**Table 6:** Best classification results (HVA±std) of various algorithms.

Methods	Caltech101	Wiki	Yale	Cornell
BSV	0.5110±0.0337 (50, 3.5896)	0.6500±0.0181 (10,3.0380)	0.9187±0.0284 (20, 3.0975)	0.6646±0.0367 (12, 5.0114)
FeaConc	0.3028±0.0225 (35, 1.8866)	0.6195±0.0185 (20, 2.0563)	0.8773±0.0250 (24, 1.1578)	0.6917±0.0602 (12, 3.2670)
KA	0.4339±0.0256 (50, 1.8555)	0.6224±0.0189 (10, 1.9641)	0.8027±0.0300 (28, 1.6784)	0.6312±0.0165 (21, 2.8715)
MVSim	0.2807±0.0209 (40, 3.9675)	0.5625±0.0179 (40, 3.2889)	0.6053±0.0475 (40, 3.0893)	0.5844±0.0487 (21, 6.3791)
MVSpec	0.2165±0.0201 (50, 3.5650)	0.6497±0.0253 (30, 4.6592)	0.4040±0.0361 (8, 2.2567)	0.5615±0.0349 (9, 4.3756)
MvSpecCE	0.2795±0.0000 (15, 254.109)	0.1988±0.1727(30, 318.7920)	0.5333±0.0000 (8, 88.5287)	0.5521±0.0000 (6, 504.64)
Cospectral	0.2764±0.0096 (30, 23.9225)	0.6360±0.0160 (45, 20.9476)	0.7453±0.0341 (20, 15.3160)	0.6646±0.0264 (15, 26.1359)
Corespectral	0.3028±0.0225 (35, 11.1016)	0.2032±0.0079 (40, 10.3300)	0.6253±0.0528 (16, 6.2484)	0.5094±0.0581 (30, 14.7143)
MSE	0.4169±0.0303 (50, 3.3677)	0.6032±0.0173 (10, 4.4454)	0.7953±0.0385 (28, 1.7592)	0.6271±0.0319 (24, 3.4674)
RMSC	0.4232±0.0246 (45, 6.8901)	0.6125±0.0219 (10, 9.3732)	0.80000±0.0377 (32, 2.2527)	0.64381±0.0274 (21, 3.3620)
LRGO-MVDR	**0.5500±0.0257 (50, 5.4184)**	**0.6637±0.0168 (15, 8.1948)**	**0.9453±0.0203 (20, 1.8526)**	**0.7177±0.0271 (9, 3.3662)**

The numbers in parentheses are the feature dimension corresponds to the best result and the training time of each algorithm, respectively.

**Fig 8:**
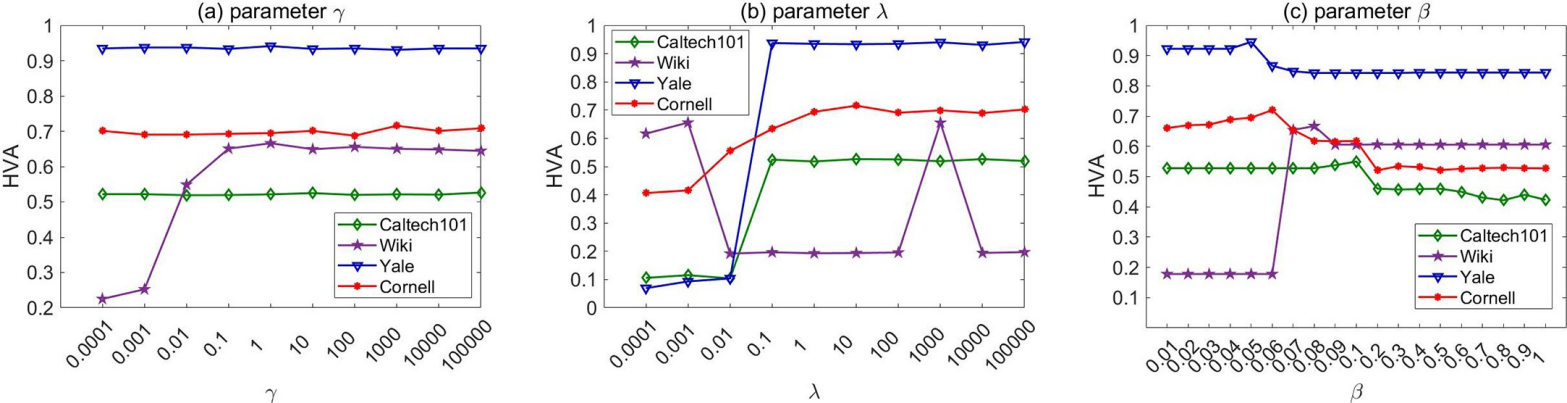
Performance of LRGO-MVDR under various parameter values for classification tasks on four datasets.

Fig 8 shows the impacts of various parameter values on the performance of our algorithm. Similar observations are made to those in the section on clustering. For the parameter *γ* which fuses the multi-view data, the performance of our method is stable across a wide range of values in most case. For *λ*, our algorithm performs better as the value of *λ* increase. This demonstrates that the noise term plays an important role. When the parameter *λ* reaches a certain value, the performance of our algorithm becomes less affected on most datasets. For the parameter *β*, we observed that our proposed algorithm achieve its best performance when the value of *β* is in a middle range such as *β*∈[0.05,0.5], which is consistent with the clustering experiments and our discussions in the section on solving for *ω*.

**Fig 9:**
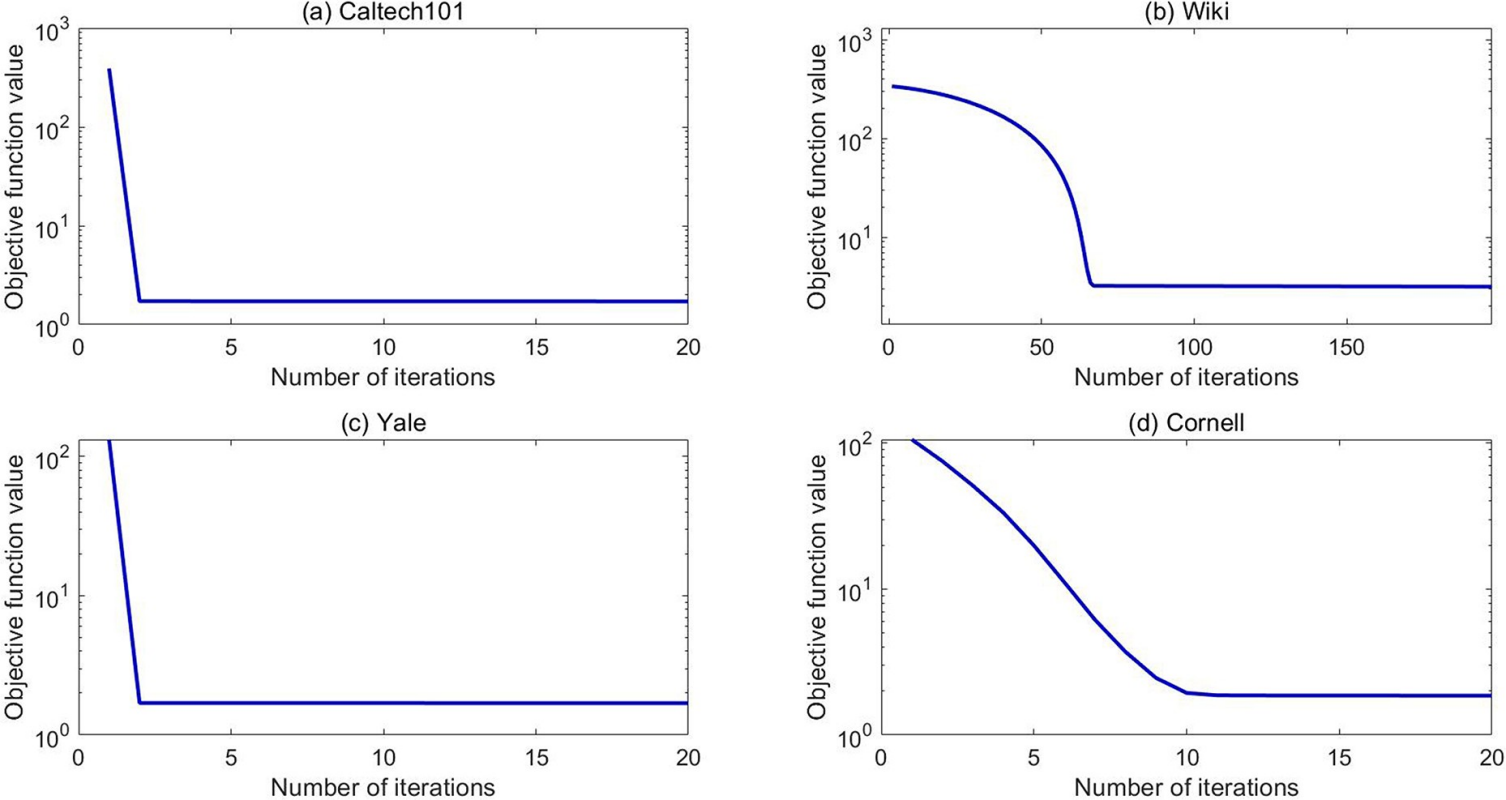
Convergence curves of LRGO-MVDR on four datasets for classification.

The convergence curves of our LRGO-MVDR algorithm on four datasets for the classification tasks are plotted in Fig 9. Similar to those in Fig 6, it is also found that the objective function rapidly deceases at the first few iterations and becomes nearly stable within 10 iterations for most datasets and 50 iterations for Wiki dataset, which empirically proves the convergence of our algorithm.

At last, we also compare the optimal classification performance of three optimization techniques to solve Eq (19) in our LRGO-MVDR. From the experimental results in Table 7, we find that LRGO-MVDR-C outperforms other two methods in terms of HVA and training time.

**Table 7:** Best classification results (HVA±std) of three methods for solving Eq (19).

Methods	Caltech101	Wiki	Yale	Cornell
LRGO-MVDR-L	0.54995924±0.0257 (50, 6.1097)	0.66367536±0.0168 (15, 8.8546)	0.94528513±0.0203 (20, 2.2008)	0.71767461±0.0271 (9, 3.8976)
LRGO-MVDR-S	0.52323286±0.0263 (50, 16.1447)	0.66153208±0.0384 (25,10.4051)	0.92404011±0.0474 (28, 7.88366)	0.70308023±0.0253 (6, 5.1217)
LRGO-MVDR-C	**0.55001860±0.0257 (50, 5.4184)**	**0.66373762±0.0168 (15, 8.1948)**	**0.94534196±0.0203 (20, 1.8526)**	**0.71771702±0.0271 (9, 3.3662)**

The numbers in parentheses are the feature dimension corresponds to the best result and the training time of each algorithm, respectively.

### Statistical significance test

To further evaluate the performance of LRGO-MVDR, a statistical significance test is adopted to determine whether the advantage of our algorithm over other methods is significant. The one tail Wilcoxon rank sum test is utilized. In this test, the null hypothesis is that LRGO-MVDR make the same performance with other methods and the alternative hypothesis is that LRGO-MVDR results in improved performance compared with other methods. For instance, to compare the performance of our algorithm with that of BSV (LRGO-MVDR vs. BSV), the null and alternative hypotheses can be defined as *H*_0_:*M*_*LRGO−MVDR*_ = *M*_*BSV*_ and *H*_1_:*M*_*LRGO−MVDR*_>*M*_*BSV*_, where *M*_*LRGO−MVDR*_ and *M*_*BSV*_ are the medians of the results that are obtained by LRGO-MVDR and BSV. In this experiment, the significance level is set to 0.05. According to the test results in Tables 8–10, the *p*-values obtained by all pairwise one-tailed Wilcoxon rank sum tests are less than 0.05. Therefore, the alternative hypotheses are accepted, and the null hypotheses are rejected in all tests. Hence, the proposed algorithm significantly outperforms the other algorithms.

**Table 8:** *p*-values of the Wilcoxon rank sum tests on clustering tasks (ACC).

	*p*-values
LRGO -MVDR vs. BSV	0.0038
LRGO -MVDR vs. FeaConc	0.0011
LRGO -MVDR vs. KA	5.7275e-06
LRGO -MVDR vs. MVSim	4.5902e-04
LRGO -MVDR vs. MVSpec	0.00019
LRGO -MVDR vs. MvSpecCE	5.7392e-05
LRGO -MVDR vs. Cospectral	1.2268e-04
LRGO -MVDR vs. Corespectral	8.2736e-05
LRGO -MVDR vs. MSE	0.0019
LRGO -MVDR vs. RMSC	0.0019

**Table 9:** *p*-values of the Wilcoxon rank sum tests on clustering tasks (NMI).

	*p*-values
LRGO -MVDR vs. BSV	1.0348e-04
LRGO -MVDR vs. FeaConc	0.0031
LRGO -MVDR vs. KA	0.0019
LRGO -MVDR vs. MVSim	3.63e-09
LRGO -MVDR vs. MVSpec	0.0019
LRGO -MVDR vs. MvSpecCE	3.5697e-09
LRGO -MVDR vs. Cospectral	0.0019
LRGO -MVDR vs. Corespectral	0.0035
LRGO -MVDR vs. MSE	0.0019
LRGO -MVDR vs. RMSC	0.0019

**Table 10:** *p*-values of the Wilcoxon rank sum tests on classification tasks (HVA).

	*p*-values
LRGO -MVDR vs. BSV	0.0479
LRGO -MVDR vs. FeaConc	0.0470
LRGO -MVDR vs. KA	0.0030
LRGO -MVDR vs. MVSim	2.8704e-07
LRGO -MVDR vs. MVSpec	2.2929e-09
LRGO -MVDR vs. MvSpecCE	3.1995e-11
LRGO -MVDR vs. Cospectral	0.0020
LRGO -MVDR vs. Corespectral	5.4697e-11
LRGO -MVDR vs. MSE	0.0039
LRGO -MVDR vs. RMSC	0.0064

## Conclusion and future work

In this paper, a graph optimization approach is proposed for multi-view dimensionality reduction. With reasonable low-rank and sparse constraints, the algorithm can effectively deal with the noise in the multi-view input data. A robust and shared graph matrix can be learned by minimizing the disagreement over the cleaned matrices. Furthermore, a weighted scheme is introduced into our algorithm to take the latent complementary information among multi-view datasets into consideration. We provide an effective iterative scheme for optimizing the LRGO-MVDR algorithm and analyze the convergence of this scheme. The experimental results on several datasets have demonstrated that the learned graph boosts the classification and clustering performance.

In future work, we will try to use multiple restarts of ADMM with random initial points, which can be regarded as a heuristic algorithm, to approximate the non-convex problem in Eq (19) [70]. Furthermore, the effectiveness of evolutionary algorithm for optimizing our approach will also be tested.

## Supporting information

S1 Appendix LRGO-MVDR_code. A file contains matlab codes of LRGO-MVDR and the datasets used in this paper.
(ZIP)
